# Piezoresistive effect in p-type 3C-SiC at high temperatures characterized using Joule heating

**DOI:** 10.1038/srep28499

**Published:** 2016-06-28

**Authors:** Hoang-Phuong Phan, Toan Dinh, Takahiro Kozeki, Afzaal Qamar, Takahiro Namazu, Sima Dimitrijev, Nam-Trung Nguyen, Dzung Viet Dao

**Affiliations:** 1 Queensland Micro and Nanotechnology Centre, Griffith University, Queensland, 4111, Australia; 2 Department of Mechanical Engineering, University of Hyogo, Hyogo, 671-2201, Japan; 3 School of Engineering, Griffith University, Queensland, 4215, Australia

## Abstract

Cubic silicon carbide is a promising material for Micro Electro Mechanical Systems (MEMS) applications in harsh environ-ments and bioapplications thanks to its large band gap, chemical inertness, excellent corrosion tolerance and capability of growth on a Si substrate. This paper reports the piezoresistive effect of p-type single crystalline 3C-SiC characterized at high temperatures, using an *in situ* measurement method. The experimental results show that the highly doped p-type 3C-SiC possesses a relatively stable gauge factor of approximately 25 to 28 at temperatures varying from 300 K to 573 K. The *in situ* method proposed in this study also demonstrated that, the combination of the piezoresistive and thermoresistive effects can increase the gauge factor of p-type 3C-SiC to approximately 20% at 573 K. The increase in gauge factor based on the combination of these phenomena could enhance the sensitivity of SiC based MEMS mechanical sensors.

Sensors based on MEMS technologies, which can withstand harsh environments, have been extensively developed and investigated recently^1^,^2^. The use of these transducers could not only improve the efficiency of systems but also predict possible failures due to hostile conditions^3^. For instance, in pipeline systems, gas flow sensors, strain sensors, and pressure sensors are required to measure the pressure level, monitor pipeline cracks, as well as detect gas leakage^4^. Additionally, in industries involving fuel combustion such as aerospace and automotive systems, temperature and pressure sensors are vital devices in the feedback control to enhance the performance of engines^5^,^6^. Among various technologies utilized in mechanical sensors, the piezoresistive effect has several advantages, such as device miniaturization, low power consumption, and simple read out circuits^7^,^8^,^9^,^10^,^11^. The piezoresistance of silicon (Si) has been deployed in a large number of applications, including inertia^12^, pressure^13^, tactile^14^, and chemical/bio sensors^15^,^16^ owing to its large gauge factor, worldwide availability and mature fabrication technologies. However, Si is not a suitable material used in harsh environments because of its relatively low energy gap of 1.12 eV and its plastic deformation at high temperature^17^.

Recent studies have paid a great attention to silicon carbide (SiC), a wide energy gap material, which possesses several superior physical properties over Si for applications under hostile conditions^18^,^19^,^20^,^21^,^22^. The piezoresistive effect of SiC has been investigated to replace Si based counterparts for high temperature applications^1^,^23^,^24^,^25^,^26^,^27^,^28^,^29^,^30^,^31^,^32^. However, one of the main obstacles for SiC material is its high cost compared to Si^1^,^25^,^26^. Researchers have aimed at growing SiC on large scale Si substrates to reduce the cost of SiC wafers as well as to ease the SiC MEMS devices fabrication processes. Among 200 poly types of crystalline SiC, 3C-SiC (or cubic SiC) is the only poly type that can be grown on a Si substrate^33^. The size of single crystalline 3C-SiC grown on Si substrates has been reported to be up to 150 mm^34^,^35^. In addition, the piezoresistance of p-type 3C-SiC grown on large scale Si wafers with a diameter of 150 mm has been reported recently, showing a gauge factor of approximately 30, which is promising for mechanical sensors^36^,^37^. However, to date, the piezoresistive effect of p-type 3C-SiC at high temperatures has not been reported. One of the main reasons that makes the characterization of the electrical properties of 3C-SiC at high temperature challenging, is the large current leakage between the SiC and Si junction at temperature above 150 °C^38^,^39^.

This paper aims to characterize the piezoresistive effect of highly doped p-type 3C-SiC at high temperatures utilizing a new measurement method in which 3C-SiC piezoresistors are heated using the Joule heating effect. We also report on the combination of the piezoresistive and themoresistive effects in 3C-SiC at high temperatures (hereinafter the combination phenomenon). A mathematical model was also proposed to extract the true gauge factor of p-type 3C-SiC from the combination of these two effects. The measurement technique proposed in this study can also be used to characterize the piezoresistive effect in other semiconductors at high temperatures, and to tune their gauge factor based on the combination phenomenon.

## Results and Discussion

### Principle of the *in situ* measurement method

**Figure 1:**
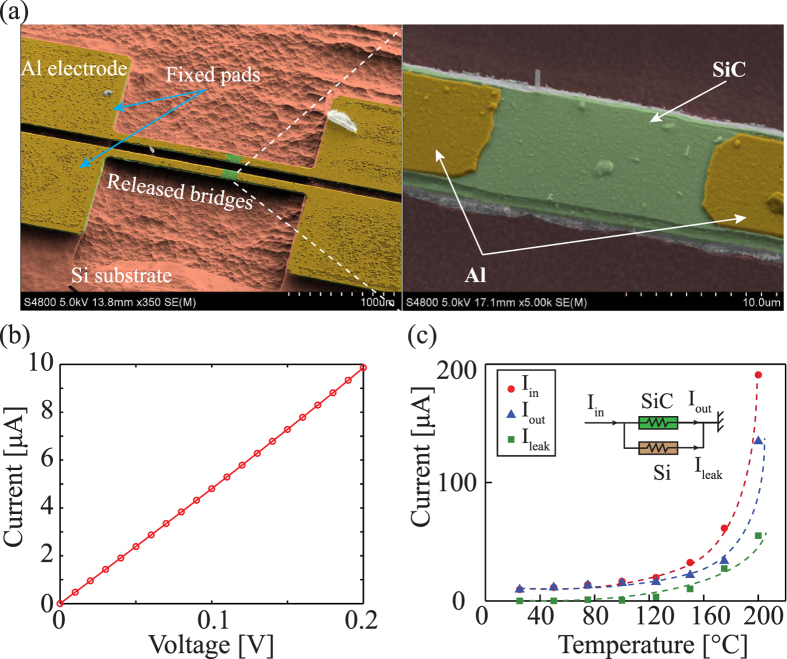
(**a**) SEM images of SiC resistors released from the Si substrate using a MEMS photolithography process; (**b**) The IV curve of a SiC resistor measured at low applied voltage to avoid the effect of Joule heating; (**c**) Measurement of the current leakage through the SiC/Si junction when the SiC/Si strip was uniformly heated at high temperatures up to 200 °C.

Figure 1(a) shows the fabricated SiC resistors on a Si strip. A SiC bridge with Al electrodes deposited and patterned on its top surface was released from the Si substrate, while its two ends were fixed to the substrate by non-released pads with a surface area of 100 *μ*m  ×  300 *μ*m. A SiC resistor with a dimension of 08 *μ*m × 12 *μ*m × 300 nm is located at the center of the released bridge.

The linear current-voltage curve of the resistor indicates that a good Ohmic contact was formed between Al and SiC, Fig. 1(b). The current leak through the SiC/Si heterojunction was found to be negligible at room temperature. The fabricated sample was then heated using a hot plate up to 200 °C. The experimental data showed that, at temperature above 100 °C the leakage current becomes significant, reaching above 10% of the current of the SiC resistor. As a consequence, the Si substrate may contribute to the measurement of the piezoresistive effect in SiC at high temperature if the whole SiC/Si strip is heated. To avoid the influence of the Si layer on the measurement of the piezoresistive effect in SiC at high temperature, there is a need to lower the temperature of the Si when heating the SiC piezoresistors. We propose the following technique to characterize the piezoresistance of the p-type 3C-SiC at elevated temperatures.

**Figure 2:**
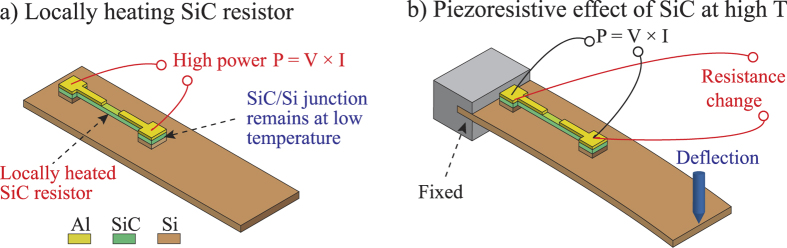
The proposed *in situ* measurement. (**a**) A high electrical power was supplied to locally raise the temperature of a released SiC resistor; (**b**) Mechanical strain was induced into the SiC resistor, while its resistance change was also measured.

As shown in Fig. 2, initially a high electrical power was supplied to the released SiC resistor to increase its temperature using the Joule heating effect. Because the Al/SiC bridge was released, the thermal conductivity through the substrate was significantly reduced. Therefore, the temperature of the Si layer remained relatively low, preventing a large leakage current occurring at high temperatures. Once the temperature of the SiC resistors reached the steady state, an external mechanical strain was then applied. Because the SiC was heated by applying an electrical power, the change in its resistance due to the piezoresistance will in turn change the applied power, and consequently change the resistance of SiC due to the thermoresistive effect. Thus, both the piezoresistive and thermoresistive effects will contribute to the modification in the electrical conductance of SiC under stress. From the change in SiC resistance due to this combined effect, the gauge factor of p-type 3C-SiC at high temperatures can be obtained. The following sections present: (i) the bending method used to characterize the piezoresistive effect; (ii) the thermoresistive effect used to monitor the temperature of the heated SiC resistor; (iii) the Joule heating effect for locally increasing the temperature of SiC; and (iv) separation of the piezoresistive effect from the combination phenomenon.

### The piezoresistive effect of 3C-SiC at room temperature

**Figure 3:**
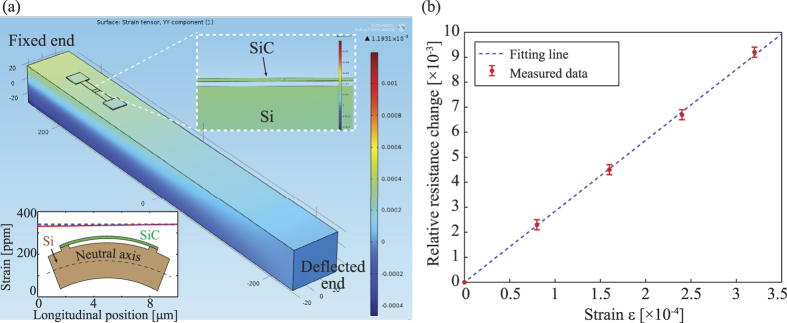
(**a**) Simulation result of the strain applied to the SiC resistor when bending the Si cantilever (inset: a comparison between the strain induced into the SiC resistor (dart line) and that of the top surface of the Si substrate (solid line)); (**b**) The relationship between the relative resistance change of the p-type 3C-SiC resistor and the applied strain at room temperature. The applied current of the multimeter was set at 500 nA, at which the Joule heating effect is considered to be negligible.

The piezoresistive effect of the released 3C-SiC was initially investigated at room temperature. The strain was induced into the SiC frame by employing the bending beam method. One end of the Si beam was fixed using a metal clamp, while the free end was deflected. The strain applied to the top surface of Si layer is:


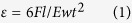


where *F* is the applied force; and *E*, *l*, *w* and *t* are the Young’s modulus, length, width, and thickness the Si cantilever, respectively. Since the released SiC bridges were fixed to the Si substrate at their two ends, we expected that the strain would be induced into the SiC resistors following the bending of the Si cantilever^40^,^41^. Finite element analysis (FEA) was then carried out using COMSOL Multiphysics™, Fig. 3(a). The simulation results indicated that the strain induced into SiC resistors was almost the same as that of the Si substrate with a difference of below 5%. Therefore, the strain applied to the fabricated SiC resistor can be approximated using equation (1).

The resistance change of SiC resistors under mechanical strain was monitored using a resistance-meter (Agilent Multimeter™). At a small applying current of 500 nA from the multimeter, the Joule heating effect in 3C-SiC resistor is considered to be negligible. Under mechanical strains varying from 00 to 350 ppm, the relative resistance change (Δ*R*/*R*) of the p-type 3C-SiC shows a linear relationship with the applied strain, Fig. 3(b). Consequently, the gauge factor of p-type 3C-SiC was calculated to be 28 at room temperature (25 °C), using the equation: *GF* = (Δ*R*/*R*)/*ε*.

The gauge factor of p-type 3C-SiC has a positive value and is at least 15 times larger than that of metals. The piezoresistive effect in p-type 3C-SiC can be qualitatively explained due to the redistribution of holes in the top valance bands. Under a mechanical strain, these bands (e.g. light holes and heavy holes) will split and warp, causing holes to move from higher energy to lower energy levels. The re-population of holes will lead to the change of their effective mass and thus their mobility, resulting in a change in SiC resistance (or conductance).

### The thermoresistive effect of 3C-SiC

**Figure 4:**
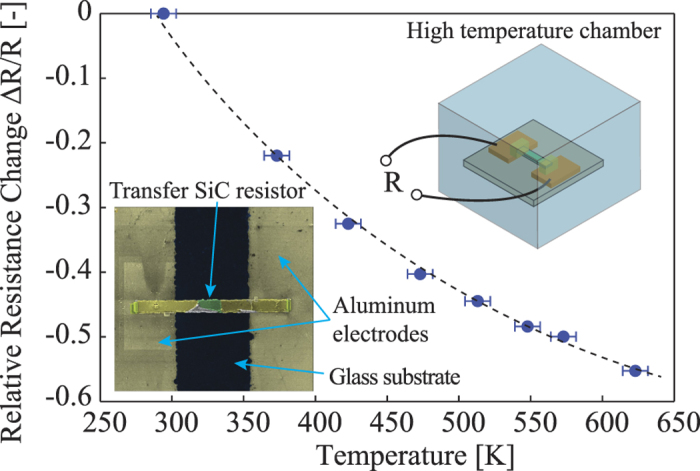
The thermoresistive effect in p-type 3C-SiC transferred on glass substrate.

Next, we characterized the thermoresistive effect of 3C-SiC to monitor the temperature of the 3C-SiC elements under Joule heating. The released SiC resistors were detached from the Si substrate using the Focused Ion Beam (FIB)^42^, as illustrated in Fig. 4 inset. Firstly, a probe was attached to the SiC bridge. Next, two ends of the released SiC resistors were diced using FIB to disconnect them from the Si substrate. Subsequently, the SiC resistors were transferred onto a glass substrate with aluminum contacts already deposited on it. Finally, tungsten was deposited onto the transferred SiC resistors to enhance the electrical contact (ESI). Because FIB was used to cut the SiC bridge on the Al area, ion bombardment was expected to have no significant impact on the SiC resistor. This assumption was confirmed as the resistance of the SiC elements before and after the FIB process showed no difference.

The thermoresistive effect was then investigated by increasing the temperature of the transferred SiC elements using a high temperature oven. It can be seen from Fig. 4 that when raising the temperature from 300 K to 600 K, the resistance of the p-type 3C-SiC decreased by approximately 50%, indicating the negative temperature coefficient of resistance (TCR) in p-type 3C-SiC. This decrease in SiC resistance is due to the thermally activated carrier concentration^43^. From the relationship between the change of SiC resistance and its temperature, the temperature of the SiC element under the Joule heating effect can be estimated.

### Joule heating in released SiC structures

The Joule heating effect was then employed to locally raise the temperatures of the SiC resistors. The physical model of the Joule heating effect in our released SiC resistor is established based on the model of suspended beam heaters, which have been extensively investigated in gas sensors^44^,^45^. Accordingly, the heating power supplied to SiC resistor is *P*_*sup*_ = *V* ×*I*, where *V* is the voltage drop across the SiC resistor and *I* is the applied current. The heat losses in the SiC bridges are caused by thermal conduction through the released beam to the anchor parts, thermal convection through the surrounding air, and thermal radiation. The heat loss through thermal conduction of the beam can be estimated as^44^: *P*_*cd*_ ∝ *A*_*cr*_*k*_*cd*_Δ*T* where *A*_*cr*_ is the cross sectional area of the SiC bridges; *k*_*cd*_ is the heat conductivity of SiC; and Δ*T* is the temperature difference between the bridge center and beam anchor, respectively. The heat loss through thermal convection to the surrounding air can be approximated as^44^: *P*_*cv*_ ∝ *A*_*sf*_*k*_*cv*_Δ*T*, where *A*_*sf*_ is the surface area of the SiC bridges, and *k*_*cv*_ is the heat transfer coefficient. Additionally, the heat loss due to thermal radiation is^46^: 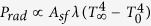, where *λ* is the Stefan–Boltzmann constant, while *T*_∞_ and *T*_0_ are the temperature of the SiC bridge and the surrounding air, respectively. At the steady state, the Joule heating effect reaches equilibrium, following the rule that the supplied power is balanced by the heat loss: *P*_*sup*_ = *V*_∞_ × *I*_∞_ = *P*_*cd*_ +*P*_*cv*_ + *P*_*rad*_. Here, the subscript ∞ indicates the steady state.

**Figure 5:**
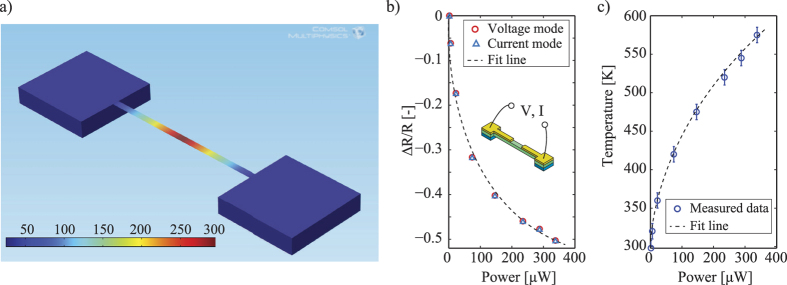
(**a**) Simulation of the Joule heating effect on released SiC resistor using Comsol Multiphysics; (**b**) The relationship between the resistance of p-type 3C-SiC resistor and the heating power at the steady state, using the current mode and voltage mode. The decrease in SiC resistance at high applied power indicates that the Joule heating effect has significantly raised the temperature of SiC resistance; (**c**) Temperature at SiC resistor calibrated from Fig. 4. The non-linearity between the applied power and temperature is considered due to thermal radiation in nano structures at high temperatures^46^.

From the above mentioned theoretical analysis, we simulated the Joule heating effect using COMSOL Multiphysics™. The temperature of the surrounding air was set to be 25 °C, while the silicon substrate was considered as the heat sink. The simulation results show that when the released SiC bridge is heated by the Joule heating effect, high temperatures mainly distributed at the center part of Al/SiC bridge and temperature gradually decreases from the center part to the anchor part. The temperature at the vicinity of the SiC/Si junction remains below 50 °C even when the temperature of the SiC resistor reaches above 300 °C, Fig. 5(a).

We conducted the Joule heating effect experiment on the released SiC resistors employing two different modes: constant current and constant voltage. Both modes showed the same result that, when the heating power was increased, the resistance of SiC decreased. Figure 5(b) presents the experimental results of the relationship between the supplied power and the SiC resistance at the steady state. The decrease in the resistance of SiC with increasing heating power obeys the thermoresistive effect presented in the previous section where increasing the temperature of the SiC element could increase its carrier concentration, thus enhance its electrical conductance. Additionally, by correlating the results in Figs 5(b) and 4, it is possible to develop the relationship between the temperature of the SiC resistor when a certain power is applied, as shown in Fig. 5(c). We also measured the leakage current through the SiC/Si junction when a large power was applied, showing that the leakage current was negligible in comparison to the current flowing through the SiC resistor (ESI). This is due to the fact that temperature rises locally at the SiC resistor while the temperature at the SiC/Si junction remains relatively low. This result is also in solid agreement with the simulation result. Due to the low heat loss caused by thermal conductance of the thin bridge, high temperatures mainly concentrated at the SiC resistor. Additionally, the temperature decreased significantly along the released bridge towards the fixed electrode pads. Therefore, the Joule heating effect can elevate the temperature of the SiC resistors and prevent the current leaking through the substrate. This result allowed us to characterize the piezoresistive effect in 3C-SiC on Si substrate *in situ*, which is presented as follows.

### Combination of the piezo- and thermo-resistive effects in locally heated 3C-SiC

Once the temperature of the SiC resistors had reached the steady state (*T*_∞_) in the Joule heating experiment, the bending method was employed to investigate the piezoresistance in SiC at high temperatures.

**Figure 6:**
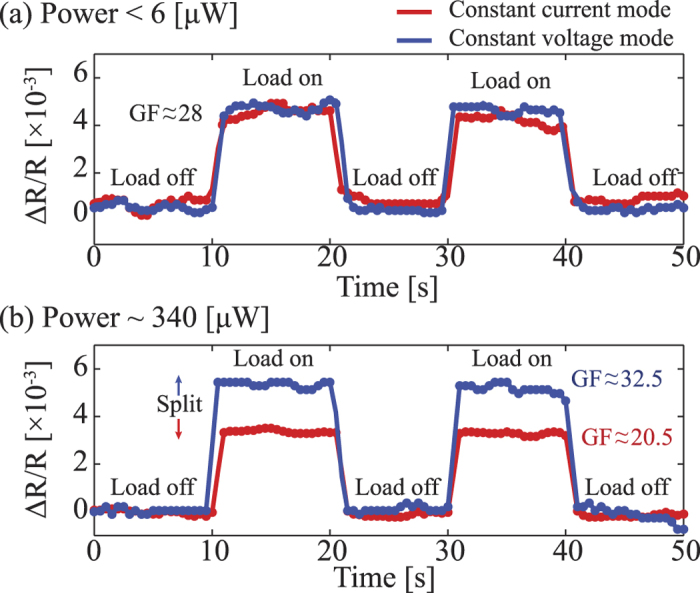
Characterization of the piezoresistive effect at high temperature using the Joule heating effect. The relative resistance change at (**a**) small applied power (**b**) high applied power. The red line shows the output of the constant current mode, while the blue line illustrates the output of the constant voltage mode.

**Figure 7:**
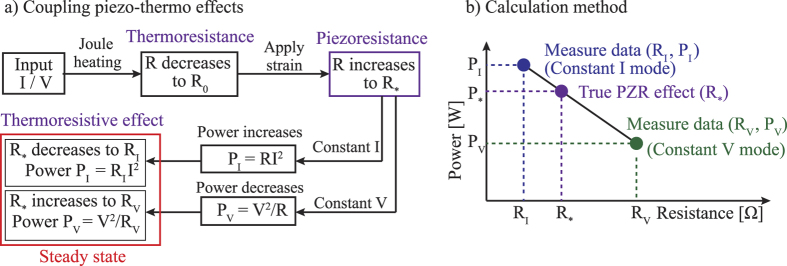
(**a**) The combination of thermoresistance and piezoresistance in the heated SiC resistors under strain; (**b**) The calculation method of the true gauge factor.

Experimental results showed that, at a low applied power (below 6 *μ*W) where the Joule heating effect was insignificant, the relative resistance changes of SiC resistor under strain in both constant current and constant voltage modes were almost the same. The gauge factor of p-type 3C-SiC was calculated to be approximately 28, which is the same as the result measured using the multimeter. However, under a high applied heating power (e.g. above 340 *μ*W), the relative resistance changes of SiC resistor under constant applied current and constant applied voltage have different values. Figure 6 shows that under the same mechanical load and heating power, the change of SiC resistance in constant voltage mode is larger than that of the constant current mode. At an applied power of 340 *μ*W which corresponds to a temperature of approximately 573 K, the gauge factor at constant voltage mode was calculated to be *GF*_*V*_ = 32.5, while at constant current mode this value was calculated to be *GF*_*I*_ = 20.5. This phenomenon was considered to be due to the combination of piezoresistance and thermoresistance in the heated SiC element under strain, as illustrated in Fig. 7(a).

The combination phenomenon can be qualitatively explained as follows. Initially, under the Joule heating effect which raises the temperature of the SiC element (T), the resistance of SiC decreases to *R*_0_ at steady state due to the thermoresistance. Next, when temperature is maintained at the steady state (T), applying the tensile strain will cause the resistance to increase to R_*_ due to the piezoresistance. Furthermore, this increase in the resistance will change the heating power supplied to the SiC element. In the case of the constant current mode, the heating power will increase as resistance increases (*P*_*I*_ = *RI*^2^), while for constant voltage mode, the heating power will decrease (*P*_*V*_ = *V*^2^/*R*), Fig. 7(a). The change of heating power in turn results in a change of resistance of the SiC piezoresistors following the thermoresistive effect. Consequently, at the steady state, the resistance of SiC under constant current mode will decrease from R_*_ to *R*_1_, while the resistance under constant voltage mode will increase from R_*_ to *R*_2_ due to the negative temperature coefficient of resistance as shown in Fig. 6(b).

Based on the combination phenomenon and the observed gauge factor at constant current and constant voltage modes, we propose a method to extract the piezoresistive effect from the influence of the thermoresistive effect at high temperature. It can be assumed from the diagram in Fig. 7 that when a mechanical strain is applied, if the power could be maintained constantly at P_*_ = *P*_0_, the temperature of SiC would be then kept constantly, and the resistance of SiC would also maintain its value of R_*_ at the steady state. Therefore, the true gauge factor of the piezoresistive effect at temperature T, should be within the gauge factors of *GF*_*I*_ and *GF*_*V*_ since the combination of thermoresistance and piezoresistance will enhance the gauge factor of SiC at the constant voltage mode; conversely the combination phenomenon reduces the gauge factor at the constant current mode.

As shown in Fig. 5(b), the resistance change shows a non-linear relationship with the applied power varying from 6 to 340 *μ*W. However, this relationship can be linearized within a smaller interval of powers by applying the Taylor series expansion to a monotonically decreasing function. As such, at each applied power in the Joule heating experiment, we measured the resistance change with the heating powers varying by ±5%. The resistance change showed a good linear relationship with the power applied in this small range, with a linear regression of 99%. Therefore, in the combination phenomenon, the heating power and resistance change can exhibit a linear relationship as the change of resistance at the steady state was relatively small (below 0.6%) resulting in a small change of heating power (below 0.6%), Fig. 7(b). A detailed explanation of the linear approximation of the relationship between the heating powers and resistances is presented in the ESI. Using this linear approximation, the following equation can be established:


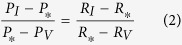


where P_*_ is the heating power at the steady state before applying strain (P_*_ = *P*_*0*_ = *V*^2^/*R*_0_ = *I*^2^*R*_0_); and *P*_*I*_ and *P*_*V*_ are the heating power at the steady state after applying strain under constant current mode, and constant voltage mode, respectively. Given that *δR* be the relative resistance change before and after applying strain, we have: *δR*_*I*_ = (*R*_*I*_ − *R*_*0*_)/*R*_*0*_, *δR*_*V*_ = (*R*_*V*_ − *R*_*0*_)/*R*_*0*_, and δR_*_ = (R_*_ − *R*_*0*_)/*R*_*0*_. Equation (2) can be written as follows:


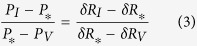


Additionally, the power differences are:


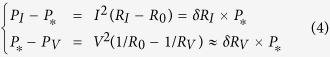


Therefore, equation (3) and equation (4) result in:


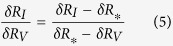


Consequently, the true relative resistance change due to the piezoresistive effect can be estimated as:


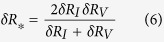


Note that as the gauge factor is the ratio of the relative resistance change to the applied strain (*GF* = *δR*/*ε*), the true gauge factor of the piezoresistive effect can be calculated based on the obtained gauge factor at the constant current and voltage modes:


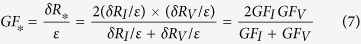


**Figure 8:**
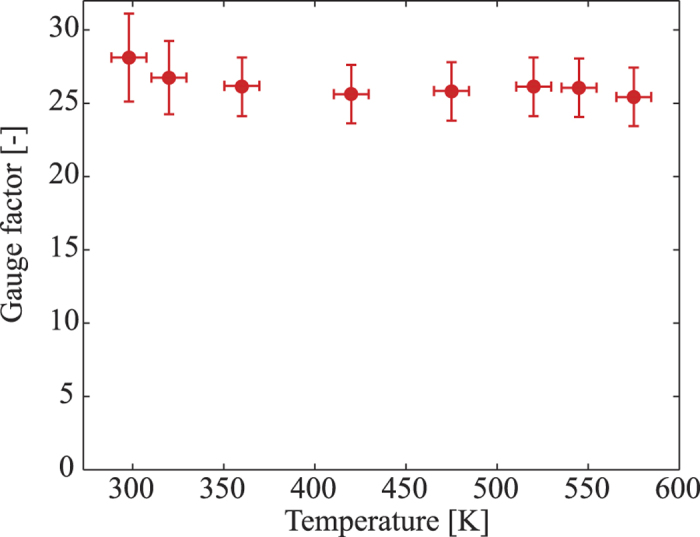
Calculated gauge factor of SiC at different heating temperatures.

The bending experiment was also performed at different applied powers varying from 6 *μ*W to 340 *μ*W, corresponding to a temperature range of 298 K to 570 K, Fig. 5(c). Additionally, using equation (7), the gauge factors of p-type 3C-SiC at different temperatures were calculated and plotted in Fig. 8. Evidently, with temperature varying from 298 K to 573 K, the gauge factor of p-type 3C-SiC was relatively stable with a smaller deviation of below 10%. This result is understandable due to the fact that the p-type 3C-SiC was relatively highly doped, making its piezoresistive effect more stable at high temperatures. The results also indicate that p-type 3C-SiC is a good candidate for applications operated at high temperatures where Si cannot be used. Additionally, the gauge factor of constant applied voltage mode was approximately 20% larger than that of the true gauge factor, indicating that by employing the piezoresistance and thermoresistance, it is possible to enhance the sensitivity of SiC based mechanical devices.

In conclusion, this work reports the piezoresistive effect in p-type 3C-SiC at high temperatures of up to 573 K, characterized using an *in situ* measurement method. With a gauge factor of above 25 at 573 K, as well as a small deviation compared to the gauge factor at room temperature, highly doped p-type 3C-SiC is a promising poly type for mechanical sensing applications used in harsh environments. We also report a physical phenomenon regarding the combination of the thermoresistive effect and the piezoresistive effect on a locally heated SiC element under mechanical strain. A physical model was also proposed to analyze this phenomenon and a calculation method to obtain the gauge factor of 3C-SiC at high temperatures was also presented. These models and method can be utilized for *in situ* characterizing of the piezoresistive effect in other semiconductor materials grown on a conductive material, as well as in tuning the gauge factor of these materials.

## Methods

### Growth of p-type single crystalline 3C-SiC

Cubic SiC films were grown on a 150 mm Si(100) wafer by using a hot-wall low pressure chemical vapor deposition (LPCVD) reactor at 1000 °C^34^,^35^. The alternating supply epitaxy approach was used to achieve single crystalline SiC film deposition with silane (SiH_4_) and propylene (C_3_H_6_) as precursors. Trimethylaluminum [(CH_3_)_3_Al, TMAl] was employed as p-type dopant to *in situ* dope the SiC wafer. The quality of the grown SiC on Si film is presented in the ESI.

### Fabrication of the released SiC resistors

After the SiC on Si wafers were grow, SiC resistors with dimensions of approximately 12 *μ*m × 8 *μ*m were patterned using a conventional MEMS photolithography process^36^. Silicon carbide on Si wafer was then diced into cantilevers with dimensions of 60 mm × 7 mm for the bending experiment. Finally, SiC resistors and Aluminum electrodes (SiC bridges with dimensions of 200 *μ*m × 8 *μ*m) were released from the Si substrate by under-etching the Si substrate using XeF_2_ gas. Additionally, all p-type 3C-SiC resistors used in this study were aligned in longitudinal [110] direction in order to obtain a large gauge factor. A detailed description of the fabrication can be found in the Electronic Supplementary Information (ESI).

## Additional Information

**How to cite this article**: Phan, H.-P. *et al*. Piezoresistive effect in p-type 3C-SiC at high temperatures characterized using Joule heating. *Sci. Rep.* **6**, 28499; doi: 10.1038/srep28499 (2016).

## Supplementary Material

Supplementary Information
