# Supplementary material for: Piezoresistive effect in p-type 3C-SiC at high temperatures characterized using Joule heating
Source: Sci Rep. 2016 Jun 28;6:28499. doi: 10.1038/srep28499 (PMC4923857; doi:10.1038/srep28499)
Supplement: Supplementary Information [file srep28499-s1.pdf]

Electronic Supplementary Information

Piezoresistive effect in p-type 3C-SiC at high temperatures characterized using Joule heating

Hoang-Phuong Phan,[*1] Toan Dinh,[1] Takahiro Kozeki,[2] Afzaal Qamar,[1] Takahiro Namazu,[2] Sima Dimitrijev,[1] Nam-Trung Nguyen,[1] and Dzung Viet Dao [1,3]

[1] *Queensland Micro-Nanotechnology Centre, Griffith University, Queensland, Australia.*
[2] *Department of Mechanical Engineering, Hyogo University, Hyogo, Japan.*
[3] *School of Engineering, Griffith University, Queensland, Australia.*
[*] *Email of corresponding author: hoangphuong.phan@griffithuni.edu.au*

1. Fabrication process

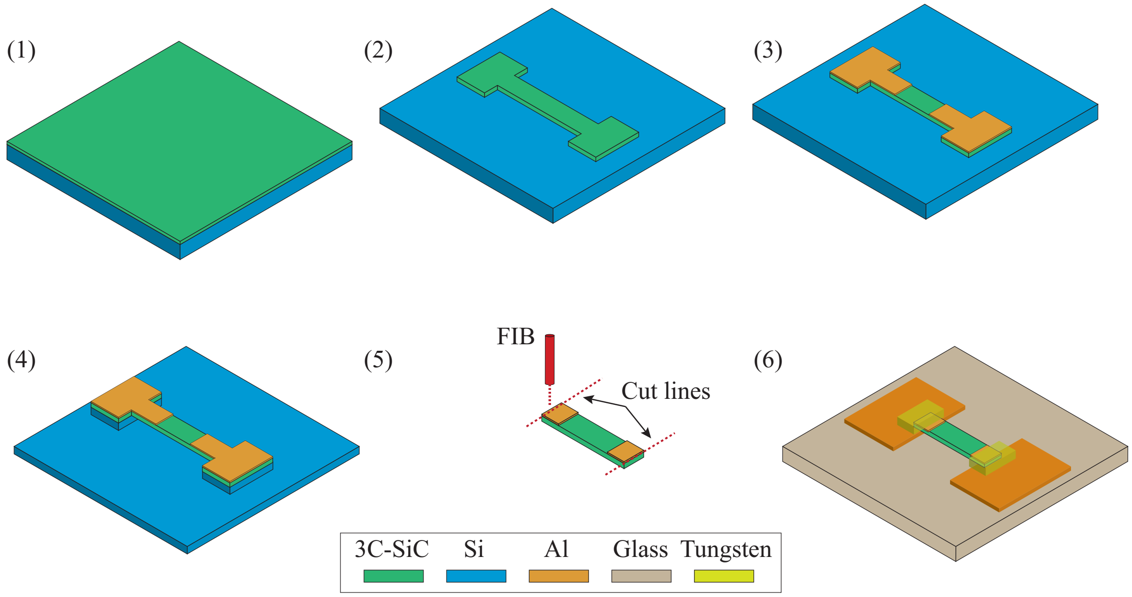

Figure 1: The fabrication process of the SiC bridges and the transferred SiC on glass substrates.

The fabrication of SiC bridges was carried out using a conventional MEMS process, Figure S1. After SiC was grown on a Si substrate (step 1), SiC resistors were patterned using ICP etching where SF_6 and O_2 were deployed as the etching gases (step 2). Next, aluminum was deposited and then etched to form the electrodes of SiC resistors (step 3). Subsequently, the SiC bridges were released using isotropic etching of the Si substrate where XeF_2 was used as the etching gas (step 4). To transfer SiC from the Si substrate to a glass substrate, Focused

Ion Beam was utilized to cut the SiC bridges (step 5). Finally, tungsten and silver epoxy were employed to make the contact between the Al electrodes and SiC resistors of the transferred samples.

2. Measurement of current leak at SiC/Si when using Joule heating effect

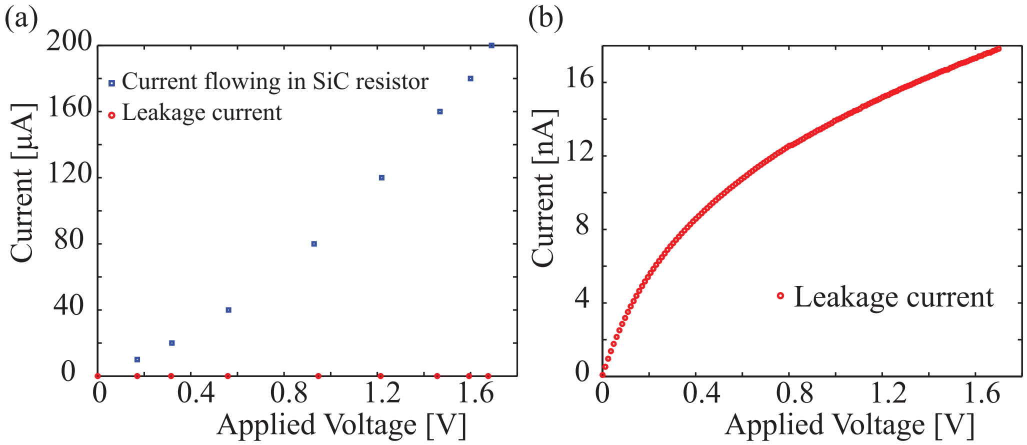

Figure 2: (a) Comparison of the current flowing in SiC resistors and the leakage current through the SiC/Si heterojunction when using the Joule heating effect. (b) The leakage current plotted in nA scale.

When increasing the temperature of SiC resistors using the Joule effect, the temperature of SiC resistor located at the center of the released bridge raised significantly, while the temperature at the vicinity of the SiC/Si junction remained at approximately at room temperature. We measured the current leak at the SiC/Si junction when increasing the electrical power applied to the SiC resistors. Evidently, at an applied voltage of 1.7 V, which corresponds to the temperature at SiC resistor of approximately 300°C, the current leak through the SiC/Si heterojunction was below 20 nA, Figure S2. Note that, as the current through SiC resistor is about 200 μA, the leakage current is negligible.

3. Approximation of the linear relationship between the applied power and resistance change in small interval

(i) In Figure 5(b) of the main article, the heating power was applied using two modes: current mode, and voltage mode, which was measured at the steady state. Initially, the heating power of the voltage mode was measured when the temperature reaching the steady state. Subsequently, the supplied current was estimated so that the same heating power could be obtained using the current mode. The results showed that with the same heating power at the steady state, both the current mode and voltage mode lead to the same resistance change. In the other words, regardless of the current or voltage modes, the same applied powers at the steady state resulted in the same temperatures (red dots coincide with the blue triangles in Figure 5(b) in the main article). Therefore, for heating powers ranging from 0 to 340 μW, the constant current and voltage modes have the same power-resistance ($P-R$) curve.

(ii) The piezoresistive effect at the current mode.
When applying a tensile strain, the SiC resistance increased from R_0 to R_* due to the piezoresistive effect. Subsequently, the heating power also increased from $P_* = R_0 I^2$ to $R_* I^2$, leading to a decrease in the SiC resistance due to the thermoresistive effect. Consequently, at the steady state, the resistance of SiC reached R_I, corresponding to a heating power of $P_I = R_I I^2$, as shown in Figure S3(a). Assuming that, at the point R_0 increased to R_*, if the applied current was modified to be $I_* = \sqrt{P_*/R_*}$, the heating power will remain at P_*. Thus, R_* will be the resistance at the steady state, since the heating power is maintained. (P_*,R_*) and (P_I, R_I) are sketched in Figure S3(b), illustrating the relationship between heating powers and resistances using the current mode.

(iii) The piezoresistive effect at the voltage mode.
Similarly, under a tensile strain, the SiC resistance increased from R_0 to R_* due to the piezoresistive effect. Subsequently, the heating power decreased from $P_* = V^2/R_0$ to V^2/R_*, leading to an increase in the SiC resistance due to the thermoresistive effect. Consequently, at the steady state, the resistance of SiC reached R_V, corresponding to a heating power of $P_V = V^2/R_V$, as shown in Figure S3(c). Assuming that, at the point R_0 increased to R_*, if the applied voltage was modified to be $V_* = \sqrt{P_* R_*}$, the heating power will remain at P_*. Thus, R_* will be the resistance at the steady state, since the heating power is maintained. (P_*, R_*) and (P_V, R_V) are plotted in Figure S3(d), illustrating the relationship between heating powers and resistances using the voltage mode. From conclusion (i), the current and voltage modes showed the same

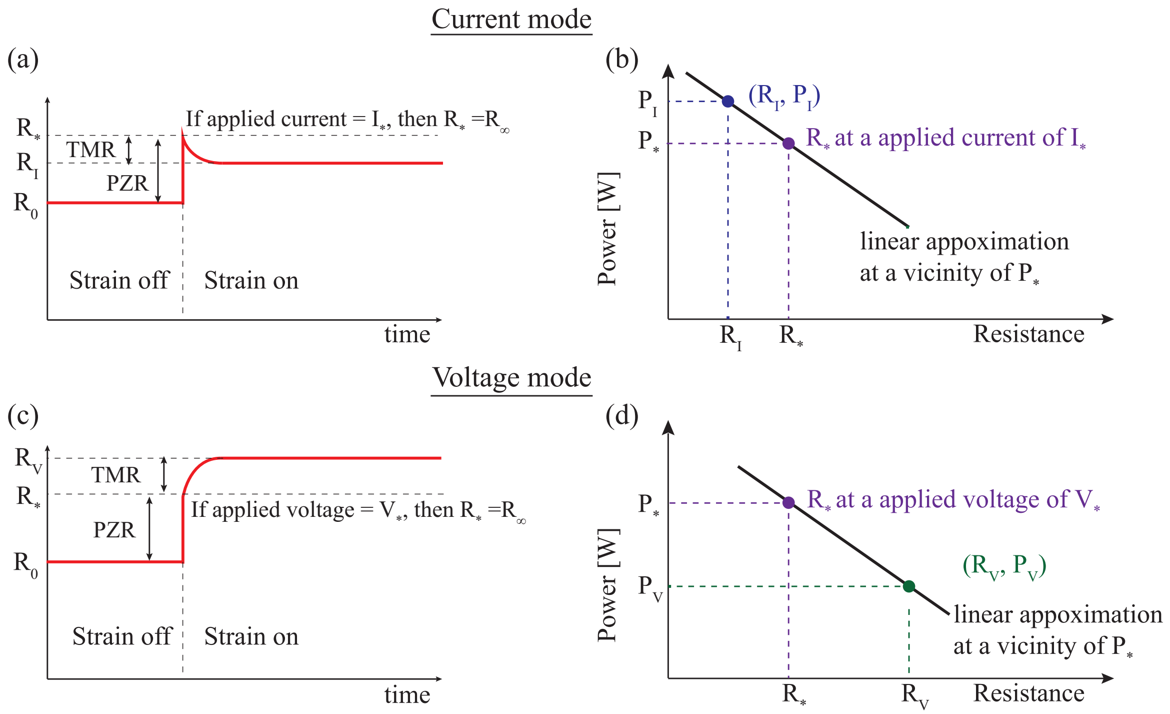

Figure 3: The interaction of the piezoresistive effect and thermoresistive effect; (a)(b) The change of resistance under current mode; (c)(d) The change of resistance under voltage mode. (The abbreviation TMR stands for the thermoresistive effect, and PZR stands for the piezoresistive effect.)

$P-R$ curve; therefore (P_I,R_I), (P_*,R_*) and (P_V,R_V) line in this $P-R$ curve which was illustrated in Fig. 7(b) in the main article.

(iv) The approximation of the linear relationship between the heating power and resistance change are presented as follows. At the steady state (T_∞), the heating power is balanced by the heat loss due to heat conductance, heat convection to air, and heat radiation [Ref. 44,46].

$$P = A(T_\infty - T_0) + B(T_\infty{}^4 - T_0{}^4) \tag{1}$$

Where A and B are constants, in which A is dominated by the heat conductance and heat convection, while B is dominated by heat radiation. Additionally, the resistance of SiC is a function of temperature, following the Arrhenius law [Ref. 43]:

$$R = Ce^{E_a/KT} \tag{2}$$

where C is a constant, E_a is the activation energy, and k is the Boltzmann constant.

The polynomial function T^4 and the exponential function $exp(E_a/kT)$ are non-linear functions of temperature (T). Therefore, the non-linear behavior of the P-R curve can be clearly observed in a large range of temperature. However, in a sufficiently small range of the applied power (or small range of temperature), the $P-R$ curve can be linearly approximated as follows. Let $P = f(T)$ and $R = g(T)$ be functions of temperature (T). In a sufficiently small interval of temperature around T_∞, it is possible to linearly approximate the heating power P and resistance R as linear functions of T using Taylor's series approximation:

$$\begin{cases} P = f(T) = f(T_\infty) + (T - T_\infty)\frac{f'(T_\infty)}{1!} + 0(T - T_\infty)^2 \\ R = g(T) = g(T_\infty) + (T - T_\infty)\frac{g'(T_\infty)}{1!} + 0(T - T_\infty)^2 \end{cases} \tag{3}$$

Where $0(R - R_\infty)^2$ is the second order of Taylor's series, and in a small interval of temperature around T_∞ (or of the applied power), this factor is negligible. In fact, this method has been widely adopted in the calculation of the temperature coefficient of resistance [Ref. 43]. In addition, from Figure 5(c) in the main article, when the heating power change is less than 1%, the temperature change is smaller than 10 K. Therefore, the polynomial function T^4 can be linearly approximated in this smaller range of temperature with a linear regression of 99.5%, as shown in Figure S4(a). Similarly, the exponential function $exp(E_a/kT)$ can also be linearly

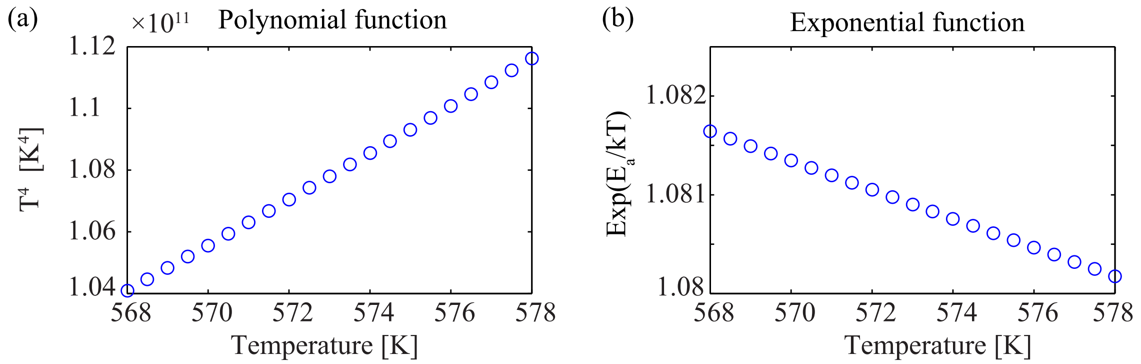

Figure 4: The polynomial and exponential functions show a good linear behavior in a small range of temperature.

approximated. As such, from the relationship between the resistance change and temperature shown in Figure 2 of the main article, the activation energy was estimated to be 38.4 meV at 573 K. Figure S4(b) shows that the linear regression of $exp(E_a/kT)$ in a temperature range of 573 ± 5 K is 99.6%. Therefore, P and R can be considered as linear functions of temperature (T) in a small interval. Thus P can be considered as a linear function of R in a small interval of the heating power.

Furthermore, our empirical results also indicated that in a sufficiently small interval of resistance change, the heating power at both current mode and voltage mode have a linear relationship with the resistances, as shown in Figure S5. This linear property has also been confirmed at the applied powers used to characterize the interaction phenomenon in the main manuscript.

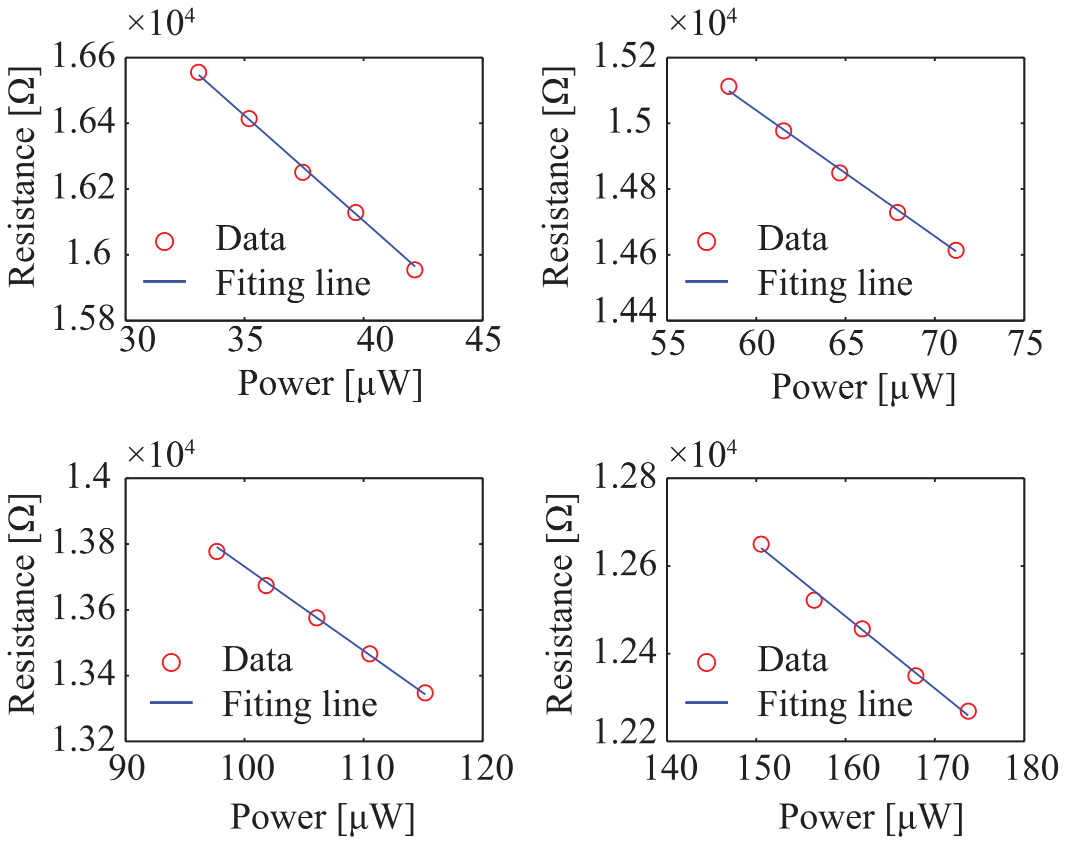

Figure 5: Experimental data showing the linear relationship between the applied power and resistance in a sufficiently small interval of resistance change (or of the applied power).

4. The properties of the p-type 3C-SiC film grown on a Si substrate

Figure S6(a) shows the photograph of a SiC on Si wafer with a diameter of 150 mm. The thickness of the wafer was measured at 300 nm, using a spectrophotometer Nanospec AFT 210^{TM}. The full-range $2\theta - \omega$ scan from x-ray diffraction (XRD) measurement indicated that the SiC film is epitaxially grown on Si(100) substrate, as shown in Fig. S6(b). Figure S6(c)

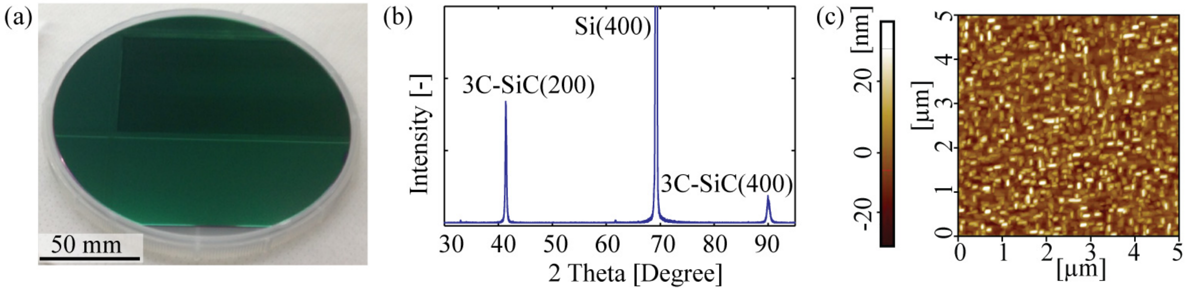

Figure 6: (a) Photograph of a 150 mm 3C-SiC on Si wafer; (b) XRD of the grown film; (c) AFM photograph of the 3C-SiC film

shows the atomic force microscopy (AFM) image of the SiC film. The roughness of the 5 μm $\times$ 5 μm area was 20 nm. The semiconductor type and carrier concentration of the 3C-SiC films were investigated using a hot probe. The polarity of the hot probe voltage indicated that SiC was p-type conductivity and the carrier concentration of p-type SiC was found to be 5×10^{18}cm^{-3} at room temperature. The Young's modulus of the 3C-SiC was found to be 330 GPa.
